# miR-146a and miR-146b promote proliferation, migration and invasion of follicular thyroid carcinoma via inhibition of ST8SIA4

**DOI:** 10.18632/oncotarget.15885

**Published:** 2017-03-03

**Authors:** Wei Ma, Xuzi Zhao, Leilei Liang, Guangzhi Wang, Yanyan Li, Xiaolong Miao, Yongfu Zhao

**Affiliations:** ^1^ Department of General Surgery, The Second Hospital of Dalian Medical University, Dalian 116044, Liaoning Province, China; ^2^ Hebei Medical University, Shijiazhuang 050017, Hebei Province, China; ^3^ Changping Hospital of Integrated Chinese and Western Medicine, Beijing 102200, Beijing Province, China

**Keywords:** follicular thyroid carcinoma, ST8SIA4, miR-146a, miR-146b, invasion

## Abstract

Follicular thyroid carcinoma (FTC) is a more aggressive form of thyroid cancer than the common papillary type. Alpha-2,8-sialyltransferase (ST8SIA) family members are expressed in various cancers and may be associated with FTC progression. In this study, we measured ST8SIA family expression in two FTC cell lines with different invasive potentials (FTC-133 and FTC-238) and Nthy-ori 3-1 cell lines, as well as FTC and normal thyroid tissues. ST8SIA4 was downregulated in the highly invasive FTC-238 cells and FTC tissues. Additionally, ST8SIA4 inhibited proliferation, migration and invasion of FTC both *in vitro* and *in vivo*. miR-146a and miR-146b were previously shown to be upregulated in thyroid carcinoma, and bioinformatics analyses indicated that miR-146a and miR-146b inhibit ST8SIA4. We found that miR-146a and miR-146b were significantly upregulated in FTC and promoted tumour progression. Furthermore, ST8SIA4 restoration decreased the invasiveness of miR-146a/b-overexpressing FTC-133 cells, and ST8SIA4 suppression reversed the effects of miR-146a/b inhibition in FTC-238 cells. We showed that miR-146a/b activated the PI3K-AKT-mTOR signalling pathway at least partially via suppression of ST8SIA4. Thus, our results demonstrate that miR-146a and miR-146b promote proliferation, migration and invasion of FTC via inhibition of ST8SIA4.

## INTRODUCTION

Thyroid cancer is the most common endocrine neoplasm; it accounts for approximately 1% of all new malignant diseases, and its annual incidence is increasing worldwide [1]. Thyroid cancer is classified into four types: papillary (85% prevalence), follicular (10% prevalence), medullary (3–4% prevalence), and anaplastic thyroid cancer (1–2% prevalence) [2, 3]. Compared with papillary thyroid cancer (PTC), follicular thyroid cancer (FTC) is more aggressive and harder to diagnose [4]. Although most FTCs can be cured with surgery, the overall 10-year survival rate of FTC is lower than that of PTC [5]. Therefore, understanding the molecular mechanisms of FTC tumours is important for developing better diagnostic strategies and improving the therapeutic outcome of FTC.

Alpha-2,8-sialyltransferase (ST8SIA I-VI) mediates the transfer of sialic acid with an alpha 2,8-linkage [6]. Previous reports demonstrated that sialic acid is involved in various biological processes, including cell-cell adhesion, immune defence, tumour cell metastasis, and inflammation [7–10]. Increasing evidence has indicated that the ST8SIA family is associated with several human tumours. For example, ST8SIA4 is involved in the development of multidrug-resistant neoplasms in acute myeloid leukaemia cells [11]. In addition, ST8SIA2 promotes the invasive properties and chemosensitivity of human hepatocellular carcinoma [12]. However, whether the ST8SIA family has biological functions in FTC is poorly understood.

MicroRNAs (miRNAs) are a class of small non-coding RNAs that activate or inhibit target genes via translational repression or mRNA degradation [13]. miRNAs regulate many biological processes, such as the cell cycle, senescence, DNA repair and tumorigenesis [14, 15]. Several reports have indicated that miRNAs are associated with thyroid cancer. For example, miR-34a promotes cell proliferation and inhibits apoptosis in PTC [16], whereas miR-200 inhibits epithelial-mesenchymal transition in anaplastic thyroid cancer [17]. However, whether miRNAs suppress ST8SIA and thereby affect the tumour characteristics of FTC remains unknown.

Activation of the PI3K-AKT-mTOR signalling pathway is implicated in the progression of thyroid cancer [18–20], and the PI3K-AKT signalling pathway is involved in the regulation of the sialyltransferase family [11]. Moreover, miRNAs have been reported to stimulate the PI3K-AKT-mTOR pathway in various diseases [21–23]. Therefore, we sought to determine whether PI3K-AKT-mTOR signalling is regulated by the ST8SIA family and miRNAs in FTC. This hypothesis has not been verified in previous studies.

In this study, we first investigated the expression and detailed role of the ST8SIA family variants in different invasive FTC cells. To elucidate the potential underlying mechanisms, we determined the role of miRNAs in the regulation of ST8SIA and FTC tumour characteristics. This work aimed to identify better preoperative markers for the diagnosis of FTC and new potential targets for its treatment.

## RESULTS

### Within the ST8SIA family, ST8SIA4 and ST8SIA6 are significantly altered in FTC

**Figure 1:**
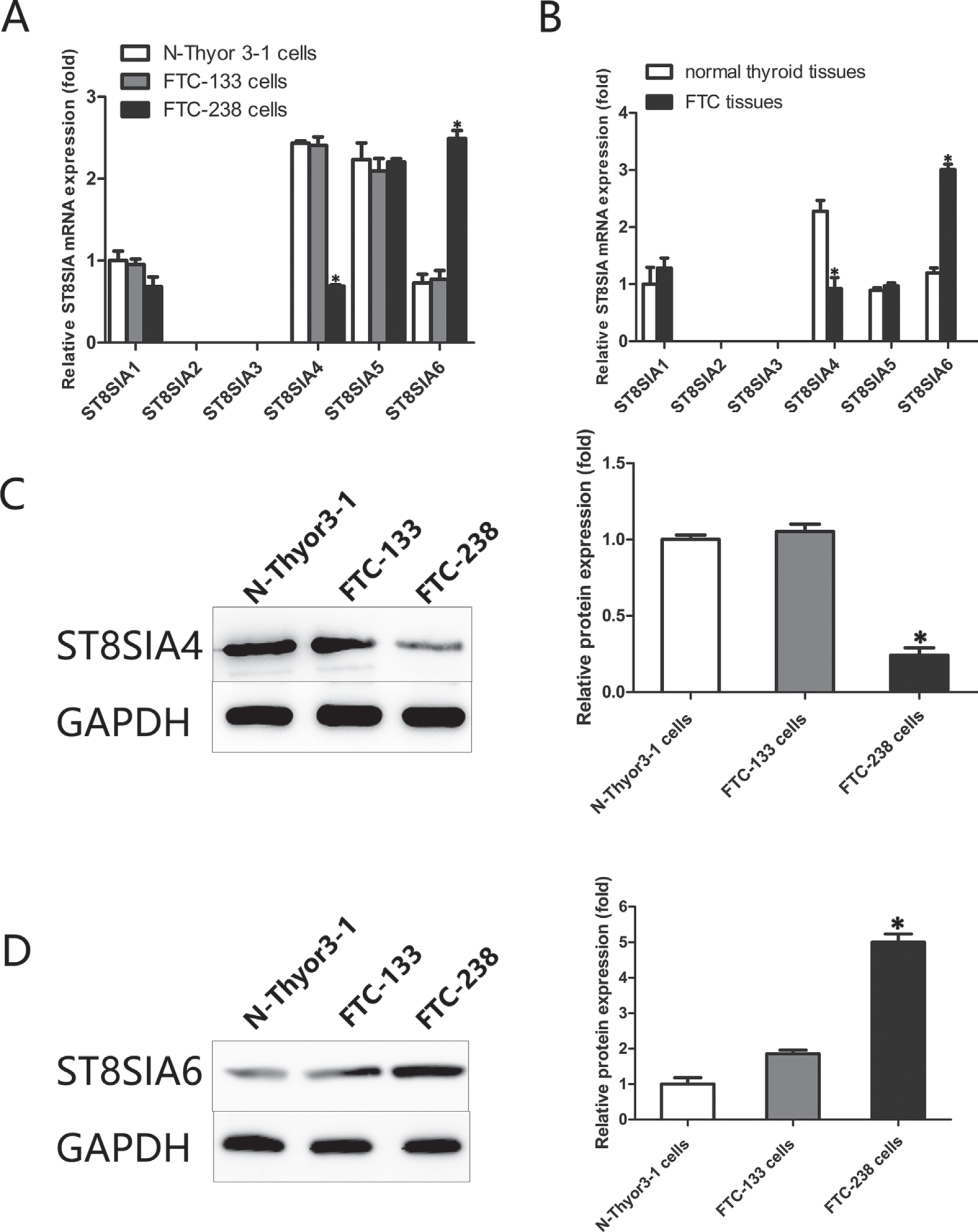
Within the ST8SIA family, ST8SIA4 and ST8SIA6 are significantly altered in FTC (**A**) mRNA levels of the ST8SIA family members in the FTC-238, FTC-133, and Nthy-ori 3-1 cells (*p < 0.05 versus FTC-133 and Nthy-ori 3-1 cells). (**B**) mRNA levels of the ST8SIA family members in the tissue samples (*p < 0.05 versus normal thyroid tissue samples). (**C**) Protein levels of ST8SIA4 and ST8SIA6 in the FTC-238, FTC-133, and Nthy-ori 3-1 cells (*p < 0.05 versus FTC-133 and Nthy-ori 3-1 cells). GAPDH served as a control. Data are shown as the mean ± SD of three repeated experiments.

Real-time PCR analyses were performed to evaluate the expression of ST8SIA family members in FTC-238 cells (highly invasive), FTC-133 cells (poorly invasive) and Nthy-ori 3-1 cells (non-invasive). As shown in Figure 1A, the expression of ST8SIA4 was significantly downregulated in the FTC-238 cells compared with that of the FTC-133 and Nthy-ori 3-1 cells (*p < 0.05). Additionally, ST8SIA6 was significantly increased in the FTC-238 cells compared with that of the FTC-133 and Nthy-ori 3-1 cells (*p < 0.05). However, in all three cell lines, ST8SIA1 and ST8SIA5 did not show significant differences in expression, and ST8SIA2 and ST8SIA3 were not expressed (*p > 0.05) (Figure 1A).

To further verify these findings in cells, we examined the expression of the ST8SIA gene family in FTC and normal thyroid tissues. As expected, mRNA expression of ST8SIA4 was significantly lower in the FTC tissues than that of the normal thyroid tissues (*p < 0.05) (Figure 1B). However, no significant differences were found in the remaining ST8SIA family members in the tissue samples, whereas the expression of ST8SIA6 was significantly higher in the FTC tissues than that of the normal thyroid tissues (*p < 0.05).

We measured ST8SIA4 and ST8SIA6 protein levels in three cell lines using western blotting (Figure 1C). Similar to the mRNA levels, ST8SIA4 protein expression was lower in FTC-238 cells than that of the FTC-133 and Nthy-ori 3-1 cells, and ST8SIA6 protein expression was higher in the FTC-238 cells than that of the FTC-133 and Nthy-ori 3-1 cells (*p < 0.05). These results indicate that ST8SIA4 and ST8SIA6 regulate FTC development.

### ST8SIA4 mediates the proliferation, migration and invasion of FTC cells both *in vitro* and *in vivo*

**Figure 2:**
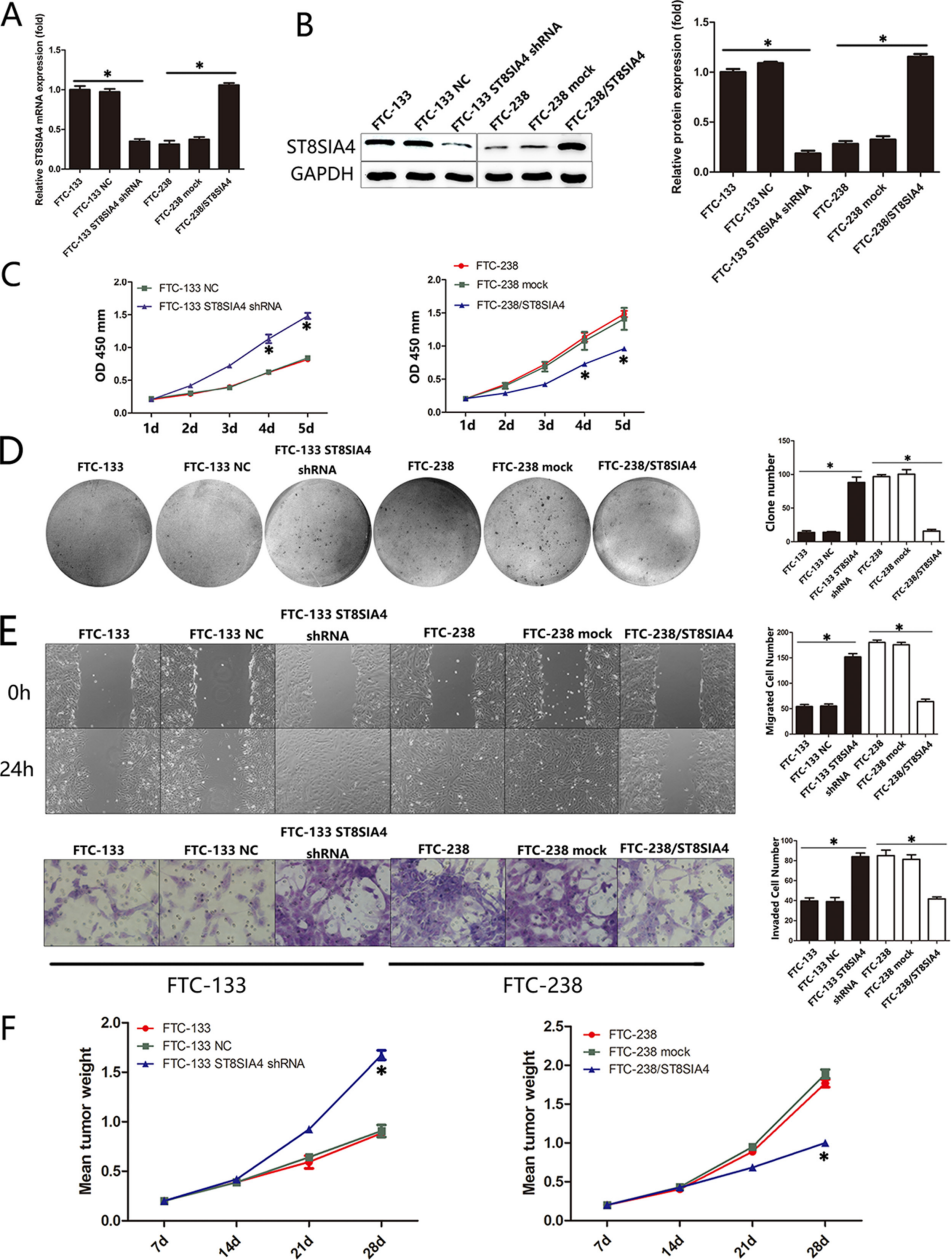
ST8SIA4 mediates the proliferation, migration and invasion of FTC cells both *in vitro* and *in vivo* FTC-133 and FTC-238 cell lines were transfected with ST8SIA4-specific shRNA or ST8SIA4 expression vector to establish the ST8SIA4-specific knockdown and overexpressing cells. (**A–B**) mRNA and protein expression of ST8SIA4 in the transfected cells. (**C**) Cell proliferation in the transfected cells. (**D**) Number of colonies formed in the transfected cells and NC cells. (**E**) Number of cells that migrated to the wound or invaded through the filter. (**F**) Transfected cells were injected subcutaneously into nude mice. The chart indicates the mean weight of the tumours after transplantation. See also Supplementary Figure 1A. Data are shown as the mean ± SD of three repeated experiments (*p < 0.05).

Based on the significant alterations in ST8SIA4 and ST8SIA6 expression in the highly invasive FTC cells, we investigated whether these two ST8SIA members could affect the tumour properties of FTC cells. First, we transfected FTC-133 and FTC-238 cell lines with ST8SIA4-specific shRNA or ST8SIA4 expression vectors to establish specific knockdown and overexpression cell lines, respectively (*p < 0.05) (Figure 2A, 2B). CCK-8 assays demonstrated that ST8SIA4 inhibition promoted FTC-133 cell proliferation, and ST8SIA4 overexpression significantly inhibited FTC-238 cell proliferation compared with that of the negative control cells (*p < 0.05) (Figure 2C). We also examined the colony formation capacity of the FTC-133 and FTC-238 cells. When ST8SIA4 was inhibited, the FTC-133 cells formed more colonies than those of the control groups, and when ST8SIA4 was overexpressed, FTC-238 cells formed fewer colonies than those of the control groups (*p < 0.05) (Figure 2D). To assess the effect of ST8SIA4 on cellular motility, we conducted wound-healing assays and transwell assays to measure the migration and invasion of FTC-133 and FTC-238 cells. Cell migration and invasion were increased in the ST8SIA4-silenced FTC-133 cells and decreased in ST8SIA4-overexpressing FTC-238 cells (*p < 0.05) (Figure 2E, Supplementary Figure 1A). However, overexpression or inhibition of ST8SIA6 did not affect the proliferation, migration and invasion of FTC cells *in vivo* (data not shown); therefore, we focused on the role of ST8SIA4 in subsequent experiments.

We further assessed whether ectopic expression of ST8SIA4 inhibited tumour growth *in vivo*. A significant decrease in tumour weight was observed in ST8SIA4-overexpressing tumours, and tumour weights were increased in the ST8SIA4-inhibited tumours compared with those of the controls (*p < 0.05) (Figure 2F). These data suggest that ST8SIA4 is an important negative regulator that mediates the proliferation, migration and invasion of FTC cells both *in vitro* and *in vivo*. These results prompted us to determine the molecular mechanisms underlying ST8SIA4 downregulation.

### miR-146a/b can directly target and inhibit the expression of ST8SIA4

**Figure 3:**
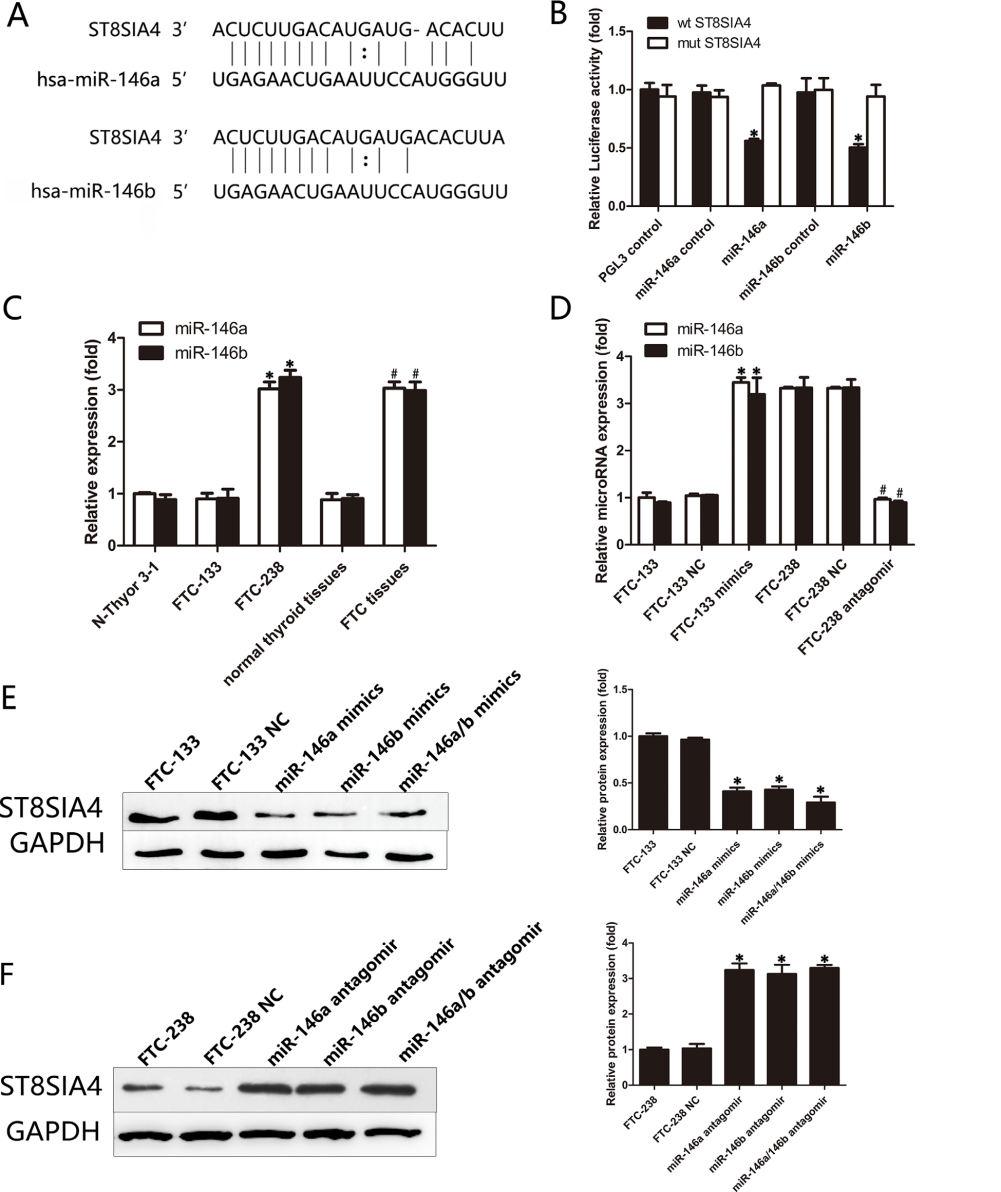
miR-146a/b can directly target and inhibit the expression of ST8SIA4 (**A**) Schematic representation of the sequence alignment of miR-146a and miR-146b and their complementary 3′UTR binding sites for ST8SIA4. (**B**) FTC-238 cells were transfected with 3′UTR-wt or 3′UTR-mut and with miR-146a/b mimics or miR-146a/b control as indicated. (**C**) Expression levels of miR-146a and miR-146b in the FTC-238 cells, FTC-133 cells, Nthy-ori 3-1 cells and tissue samples. miR-146a and miR-146b expression was normalized using U6 expression (*p < 0.05 versus Nthy-ori 3-1 and FTC-133 cells; ^#^*p* < 0.05 versus normal thyroid tissue samples). (**D**) Expression of miR-146a and miR-146b in the FTC-133 cells transfected with the mimics or in the FTC-238 cells transfected with the antagomirs (*p < 0.05 versus FTC-133 and FTC-133 NC cells; ^#^*p* < 0.05 versus FTC-238 and FTC-238 NC cells). (**E**) Protein levels of ST8SIA4 in the FTC-133 cells transfected with miR-146a and/or miR-146b mimics (*p < 0.05 versus NC cells). (**F**) Protein levels of ST8SIA4 in the FTC-238 cells transfected with miR-146a and/or miR-146b antagomirs (*p < 0.05 versus NC cells). Data are shown as the mean ± SD of three repeated experiments.

miRNAs are potent gene regulators in a wide range of diseases. Because ST8SIA4 may be suppressed by miRNAs, we searched for potential targets of ST8SIA4 using the bioinformatics algorithms TargetScan (http://www.targetscan.org), miRanda (http://www.microrna.org/microrna/home.do) and mirbase (http://www.mirbase.org). We identified several miRNAs that may target ST8SIA4, including miR-146a, miR-146b, miR-664-3p, miR-216a-5p, and miR-380-3p (data not shown). Notably, miR-146a and miR-146b were reportedly upregulated in thyroid cancer and promote the progression of thyroid cancer [24–26]. Figure 3A shows the sequence of the miR-146a and miR-146b seed regions and their 3′UTR target sites for ST8SIA4. To confirm that ST8SIA4 is a direct target of miR-146a and miR-146b in FTC, we transfected a luciferase fusion construct containing either the wild-type or mutated ST8SIA4 3′UTR into FTC-238 cells. As shown in Figure 3B, the luciferase activity of the wild-type (WT) 3′UTR reporter gene was significantly decreased, whereas the luciferase activity of the mutant reporter gene was not affected (*p < 0.05). These data indicate that miR-146a/b binds to the 3′UTR of the ST8SIA4 gene.

To further explore the expression of miR-146a/b in FTC, we examined the expression of miR-146a and miR-146b in cells and tissues (Figure 3C). As shown in Figure 3C, miR-146a and miR-146b levels increased in the FTC-238 cells compared with those of the FTC-133 or Nthy-ori 3-1 cells (*p < 0.05). In addition, the expression of miR-146a and miR-146b was higher in the FTC tissues than that of the normal thyroid tissues (^#^*p* < 0.05) (Figure 3C). We further examined the expression of miR-146a and miR-146b in FTC-133 cells transfected with miR-146a or miR-146b mimics and the FTC-238 cells transfected with miR-146a or miR-146b antagomirs (Figure 3D).

The ST8SIA4 protein levels were downregulated in the FTC-133 cells transfected with miR-146a and/or miR-146b mimics compared with those of the NC cells (*p < 0.05) (Figure 3E). Moreover, the ST8SIA4 protein levels were increased in the FTC-238 cells transfected with miR-146a and/or miR-146b antagomirs compared with those of the NC cells (*p < 0.05) (Figure 3F). Taken together, these results demonstrate that miR-146a and miR-146b are negative regulators of ST8SIA4 in FTC.

### miR-146a/b regulates the proliferation, migration and invasion of FTC-133 cells and FTC-238 cells both *in vitro* and *in vivo*

**Figure 4:**
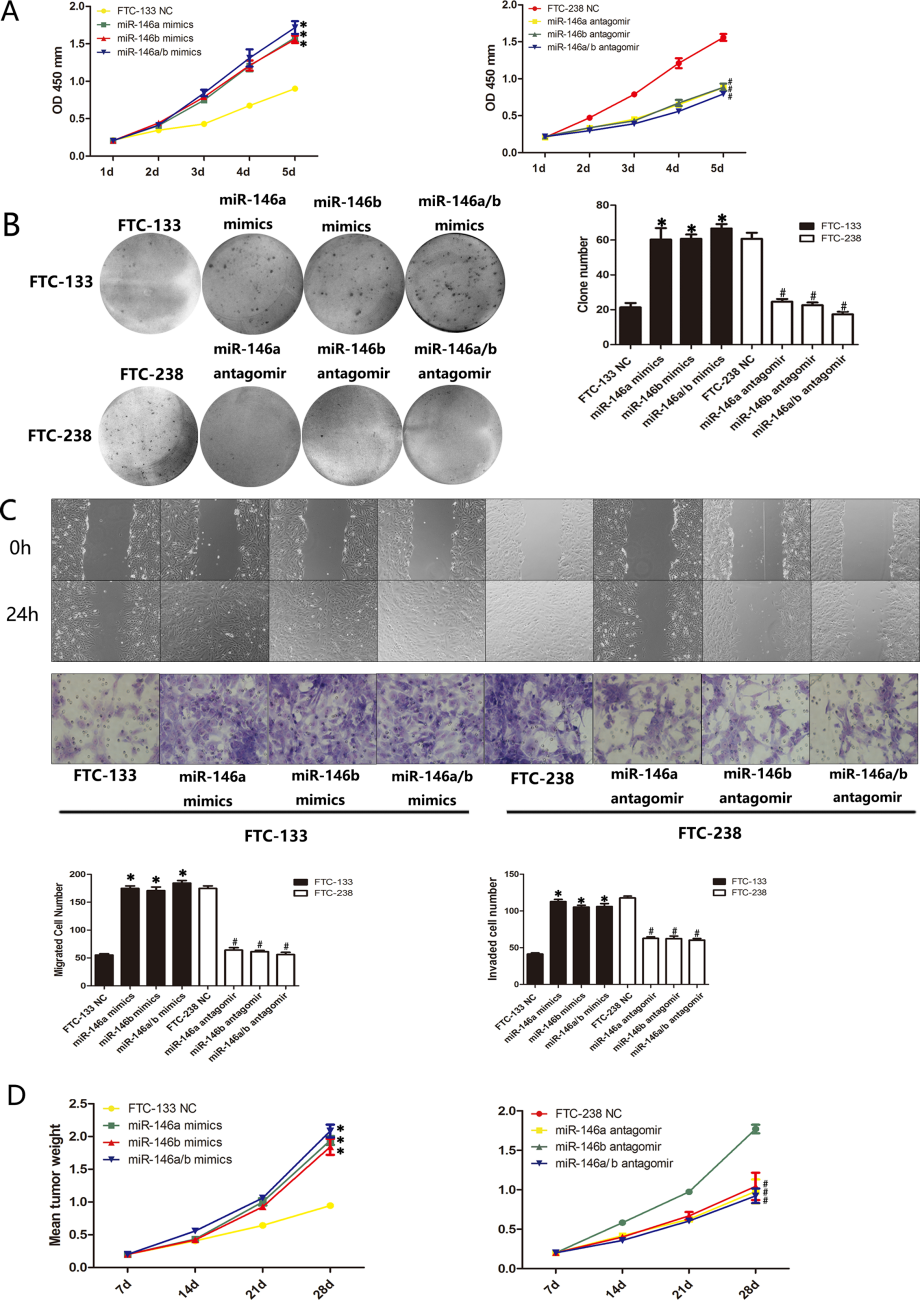
miR-146a/b regulates the proliferation, migration and invasion of FTC-133 cells and FTC-238 cells both *in vitro* and *in vivo* FTC-133 cells transfected with miR-146a and/or miR-146b mimics and FTC-238 cells transfected with miR-146a and/or miR-146b antagomirs. (**A**) Cell proliferation of the transfected cells and NC cells. (**B**) Number of colonies formed in the transfected cells and NC cells. (**C**) Number of cells that migrated to the wound or invaded through the filter. (**D**) Transfected cells were injected subcutaneously into nude mice. The weight of the tumours after transplantation. See also Supplementary Figure 1B. Data are shown as the mean ± SD of three repeated experiments (*p < 0.05 versus FTC-133 NC cells; ^#^*p* < 0.05 versus FTC-238 NC cells).

To further elucidate the role of miR-146a/b in FTC, we investigated the proliferation, migration and invasion of miR-146a/b-silenced FTC-238 cells or miR-146a/b-overexpressing FTC-133 cells. CCK-8 assays demonstrated that miR-146a and/or miR-146b overexpression significantly promoted FTC-133 cell proliferation relative to that of the NC cells, whereas miR-146a and/or miR-146b inhibition reduced FTC-238 cell proliferation compared with that of the NC cells (*p < 0.05, ^#^*p* < 0.05) (Figure 4A). The colony formation assays indicated that FTC-133 cells overexpressing miR-146a and/or miR-146b formed more colonies than those of the control groups, and miR-146a- and/or miR-146b-inhibited FTC-238 cells formed fewer colonies than those of the control groups (*p < 0.05, ^#^*p* < 0.05) (Figure 4B). As shown in Figure 4C, miR-146a and/or miR-146b overexpression increased the migration and invasion of FTC-133 cells, whereas the inhibition of miR-146a and/or miR-146b reduced the migration and invasion of FTC-238 cells (*p < 0.05, ^#^*p* < 0.05).

Consistent with the results of the above assays, the nude mouse xenograft model showed an increase in tumour weight relative to that of the NC cells when the FTC-133 cells were transfected with miR-146a and/or 146b mimics (*p < 0.05) (Figure 4D) and a decrease in tumour weight relative to the NC cells when the FTC-238 cells were transfected with miR-146a and/or miR-146b antagomirs (^#^*p* < 0.05) (Figure 4E, Supplementary Figure 1B). These data suggest that miR-146a/b promotes the proliferation, migration and invasion of FTC both *in vitro* and *in vivo*.

### ST8SIA4 suppresses the effects of miR-146a/b in FTC

**Figure 5:**
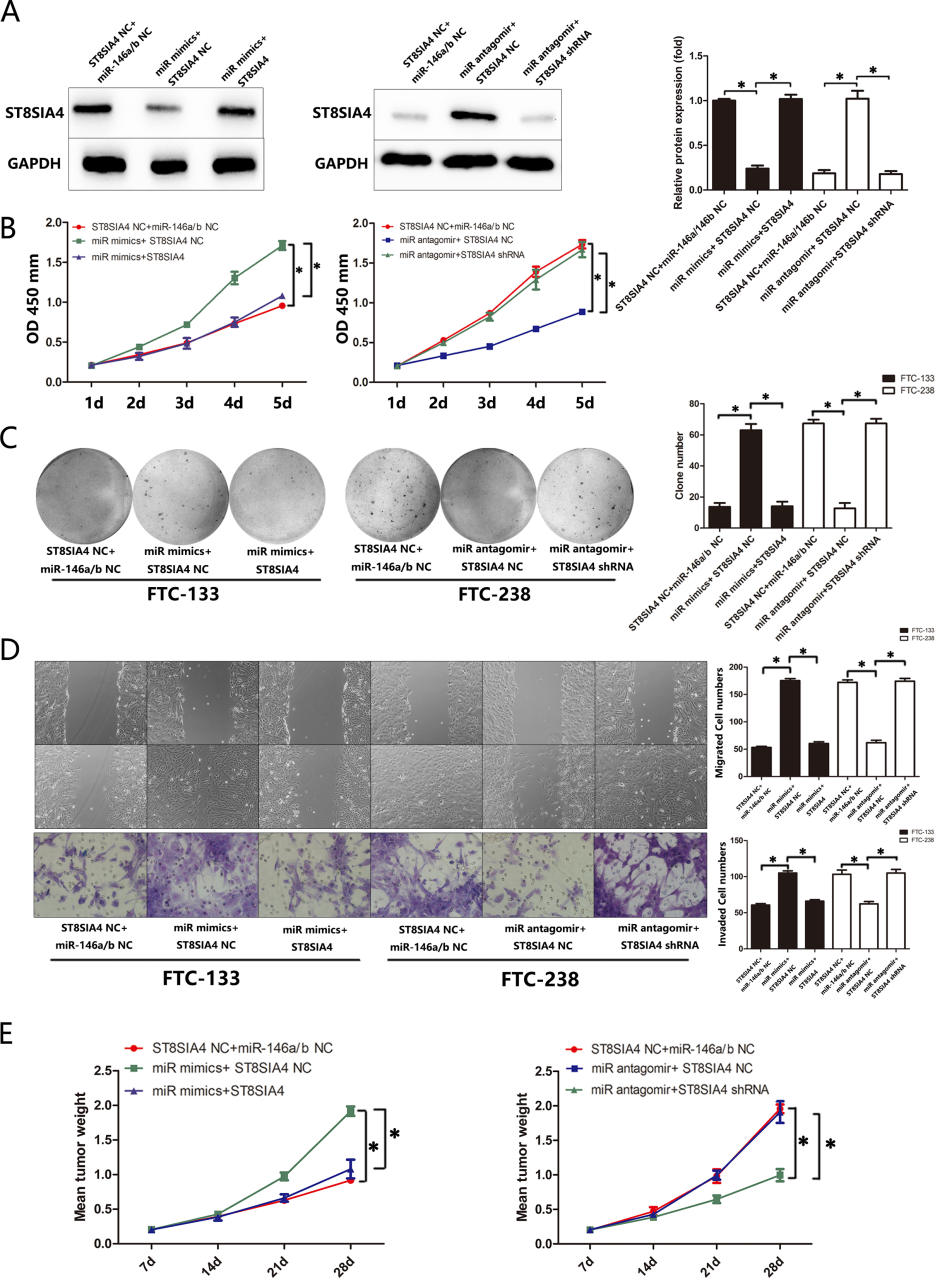
ST8SIA4 suppresses the effects of miR-146a/b in FTC FTC-133 cells were co-transfected with miR NC and ST8SIA4 NC, miR-146a/b mimics and ST8SIA4 NC, or miR-146a/b mimics and ST8SIA4 shRNA. FTC-238 cells were co-transfected with miR NC and ST8SIA4 NC, miR-146a/b antagomirs and ST8SIA4 NC, or miR-146a/b antagomirs and ST8SIA4. (**A**) Protein levels of ST8SIA4 in the transfected cells. (**B**) Cell proliferation of the transfected cells and NC cells. (**C**) Number of colonies formed in the transfected cells and NC cells. (**D**) Number of cells that migrated to the wound or invaded through the filter. (**E**) Weight of the tumours after transplantation. See also Supplementary Figure 1C. Data are shown as the mean ± SD of three repeated experiments (*p < 0.05).

To investigate whether the effects of miR-146a/b in FTC are mediated by ST8SIA4, we rescued ST8SIA4 expression in the miR-146a/b-overexpressing FTC-133 cells and suppressed ST8SIA4 expression in the miR-146a/b-inhibited FTC-238 cells. As shown in Figure 5A–5D, ST8SIA4 restoration decreased the proliferation, migration and invasion of miR-146a/b-overexpressing FTC-133 cells and partially decreased the migration and invasion capacity of these cells compared with those of the NC cells (*p < 0.05). Furthermore, ST8SIA4 suppression partially increased the proliferation, migration and invasion of miR-146a/b-inhibited FTC-238 cells compared with those of the NC cells (*p < 0.05). Importantly, the FTC-133 cells transfected with the ST8SIA4 expression vector reversed the tumour growth induced by miR-146a/b *in vivo*, whereas the FTC-238 cells transfected with the ST8SIA4-specific shRNA reversed the tumour growth inhibitory effect of miR-146a/b inhibition *in vivo* (Figure 5E, Supplementary Figure 1C). These results suggest that ST8SIA4 is an important participant in the regulation of miR-146a/b and suppresses FTC proliferation, migration and invasion.

### miR-146a/b is inversely correlated with ST8SIA4 in FTC and associated with the activation of the PI3K-AKT-mTOR signalling pathway

**Figure 6:**
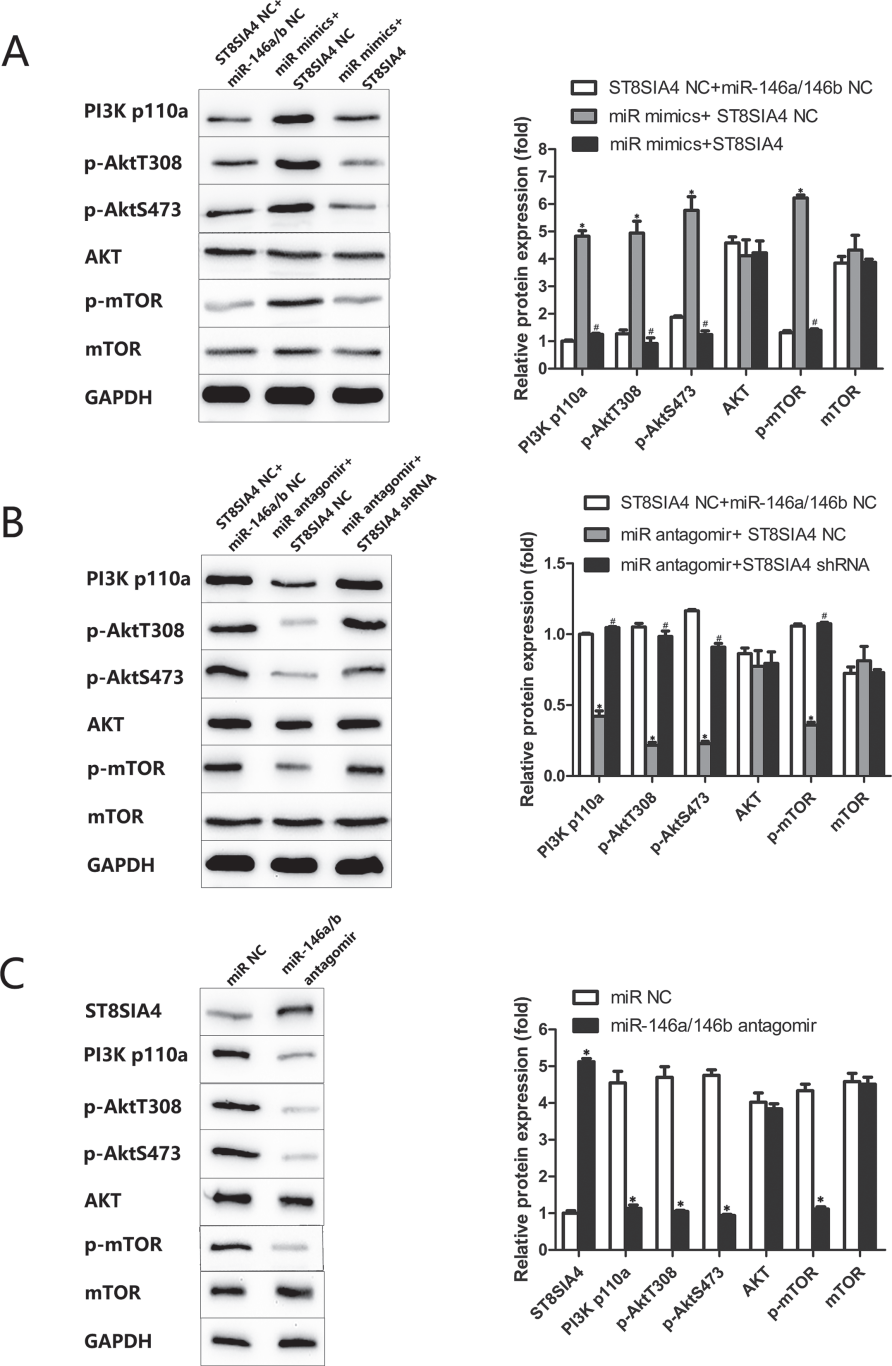
miR-146a/b is inversely correlated with ST8SIA4 in FTC and associated with the activation of the PI3K-AKT-mTOR signalling pathway FTC-133 cells were co-transfected with miR NC and ST8SIA4 NC, miR-146a/b mimics and ST8SIA4 NC, or miR-146a/b mimics and ST8SIA4 shRNA. FTC-238 cells were co-transfected with miR NC and ST8SIA4 NC, miR-146a/b antagomirs and ST8SIA4 NC, or miR-146a/b antagomirs and ST8SIA4. (**A**) Protein levels of PI3K p110α, p-Akt T380, p-Akt S473, AKT, p-mTOR and mTOR in the transfected FTC-133 cells (*p < 0.05 versus NC cells; ^#^*p* < 0.05 versus miR-146a/b mimics and ST8SIA4 NC). (**B**) Protein levels of the above molecular markers in the transfected FTC-238 cells (*p< 0.05 versus NC cells; ^#^*p* < 0.05 versus miR-146a/b antagomirs and ST8SIA4 NC). (**C**) Protein levels of the above molecular markers and ST8SIA4 in the xenograft tumour tissue with miR-146a/b antagomirs (*p < 0.05 versus NC cells).

Previous studies have shown that the PI3K-AKT-mTOR signalling pathway is regulated by the sialyltransferase family and miRNAs in various cancers, including thyroid cancer [11, 18–23]. Therefore, we evaluated the ability of miR-146a/b and ST8SIA4 to regulate the PI3K-AKT-mTOR signalling pathway both *in vitro* and *in vivo*. As shown in Figure 6, PI3K p110α, phosphorylated Akt (p-Akt T380, p-Akt S473) and phosphorylated mTOR (p-mTOR) were increased in miR-146a/b-overexpressing FTC-133 cells and partially decreased after ST8SIA4 restoration (*p < 0.05, ^#^*p* < 0.05) (Figure 6A). Furthermore, ST8SIA4 suppression partially increased the levels of PI3K p110α, p-Akt T380, p-Akt S473 and p-mTOR in miR-146a/b-inhibited FTC-238 cells compared with those of the NC cells (*p < 0.05, ^#^*p* < 0.05) (Figure 6B). The protein levels of PI3K p110α, p-Akt T380, p-Akt S473, p-mTOR and ST8SIA4 were decreased in the xenograft tumour tissues transfected with miR-146a/b antagomirs (*p < 0.05) (Figure 6C). Additionally, total AKT and mTOR protein levels did not change in the three experiments (*p > 0.05, ^#^*p* < 0.05) (Figure 6A–6C). These results suggested that miR-146a/b activates the PI3K-AKT-mTOR signalling pathway, which is suppressed by ST8SIA4.

## DISCUSSION

FTC is the second most common malignancy originating from thyroid follicular cells. In this study, we provided the first evidence of the following four findings: (1) ST8SIA4 expression is lower in FTC tissues and cultured highly invasive FTC cells than that in normal tissues and cells and suppressed the proliferation, migration and invasion of FTC both *in vitro* and *in vivo*; (2) miR-146a/b is significantly upregulated in the highly invasive FTC cells, which are direct targets of ST8SIA4; (3) miR-146a/b inhibition attenuates the proliferation, migration and invasion of FTC by repressing ST8SIA4 both *in vitro* and *in vivo*; and (4) miR-146a/b suppresses ST8SIA4 in FTC partly via regulation of the PI3K/AKT/mTOR signalling pathway. To the best of our knowledge, this is the first study to explore the inhibitory role of ST8SIA4 in regulating the tumour characteristics of FTC and the relationship of ST8SIA4 and miRNAs in FTC.

Sialyltransferases catalyse the transfer of sialic acid to proteins and lipids and participate in the synthesis of the core structure of oligosaccharides [27]. Native sialylation is critical for the function of therapeutic proteins because it affects the physical, chemical and immunogenic properties of glycoproteins [28]. Importantly, altered sialylation is a hallmark of cancer, and abnormally expressed sialylated glycans are biomarkers of cancer [29]. In this study, we found that the expression of ST8SIA4, a type of sialyltransferase, was lower in highly invasive FTC cells (FTC-238 cells) than that in minimally invasive FTC cells (FTC-133 cells). Then, we performed functional analyses and found that ST8SIA4 downregulation may contribute to the aggressive properties of FTC. These findings prompted us to further explore the detailed mechanisms underlying ST8SIA4 downregulation in FTC.

miRNAs are potent gene regulators that have been implicated in multiple pathophysiological processes and may play a crucial role in the progression of thyroid cancer [17, 30–32]. Thus, we sought to determine whether ST8SIA4 expression is controlled by miRNAs. Bioinformatics analysis and luciferase activity assays confirmed miR-146/b binding to the 3′UTR of the ST8SIA4 mRNA. According to these results, we suggest that miR-146a and miR-146b are potential regulators of ST8SIA4.

Previous reports have shown that miR-146a and miR-146b are positively regulated in thyroid cancer [24, 26, 33]. We found that miR-146a and miR-146b promoted the tumour characteristics of FTC both *in vivo* and *in vitro*. Importantly, our gain- and loss-of-function analyses showed that the effects of miR-146a/b knockdown in the FTC-238 cells were reversed by inhibition of ST8SIA4 and vice versa. Thus, miR-146a/b suppresses ST8SIA4 in FTC, which consequently promotes the proliferation, migration and invasion of FTC cells.

Ma *et al*. found that the PI3K-AKT pathway is located downstream of ST8SIA4 [11]. In this study, we further verified that the PI3K-Akt-mTOR signalling pathway is involved in the regulation of miR-146a/b and ST8SIA4 in FTC. In addition, the expression of the PI3K-AKT-mTOR pathway mimics the expression of miR-146a/b in FTC-238 cells and FTC-133 cells, which are negatively regulated by ST8SIA4. Therefore, we suggest that PI3K-Akt-mTOR pathway activation is correlated with the tumour properties of FTC.

A single miRNA may target several transcripts [34]; therefore, additional miR-146a and miR-146b targets may be involved in regulating the tumour characteristics of FTC. PTTG1, which is targeted by miR-146a and miR-146b, is also regulates thyroid cancer [35]. However, PTTG1 expression was not significantly altered in FTC-133 and FTC-238 cells when miR-146a and miR-146b were inhibited (data not shown). PTEN, another target of miR-146a and miR-146b, may also suppress thyroid cancer [36–38]. However, whether this gene participates in FTC progression requires further investigation.

In this study, we found that miR-146a and miR-146b promote the proliferation, migration and invasion of FTC via inhibition of ST8SIA4 and regulation of the PI3K-AKT-mTOR signalling pathway. Our findings further elucidate the molecular mechanisms underlying FTC progression and provide candidate targets for the prevention and treatment of FTC. Although additional studies are needed to explore these therapeutic targets, we confirmed that ST8SIA4 and miR-146a/b play key roles in FTC.

## MATERIALS AND METHODS

### Cell culture

Human thyroid cell lines (FTC-133, FTC-238, and Nthy-ori 3-1) were purchased from Jennio Biotech Co. (Guangdong, Guangzhou, China). The cells were cultured in Dulbecco's modified Eagle's medium (DMEM) (Gibco, Grand Island, NY, USA) supplemented with antibiotics (100 U/ml 1×penicillin/streptomycin, Gibco) and 10% heat-inactivated foetal bovine serum (Gibco). The cells were incubated at 37°C in a humidified atmosphere containing 5% CO_2_.

### Tissue samples

After informed consent was obtained, resected thyroid cancer tissue samples were collected from 110 patients diagnosed with FTC (48 men and 62 women; median age, 58; range, 37–76). Normal thyroid tissue samples were collected from 110 patients diagnosed with thyroid nodules (41 men and 69 women; median age, 41; range, 24–69). All patients underwent surgical resection between January 2012 and June 2015 at the Second Affiliated Hospital of Dalian Medical University. The investigation and informed consent protocol were certified and approved by the Ethics Committee of the Second Affiliated Hospital of Dalian Medical University. The pathological diagnosis of the collected samples was confirmed according to the Union for International Cancer Control (UICC) criteria. The clinicopathological data were extracted from the patient's medical records. None of the patients had received chemotherapy or radiation therapy. The collected tissues were snap-frozen in liquid nitrogen and stored at −80°C until use.

### RNA isolation and real-time PCR

Total RNA was isolated from the cell lines using an RNeasy Mini Kit (Qiagen, Valencia, CA), and cDNA was synthesized using a QuantiTect Reverse Transcription Kit (Qiagen, Valencia, CA) according to the manufacturers’ protocol. For the mRNA expression analysis, 3 μg of total RNA was converted into cDNA using M-MLV Reverse Transcriptase (Invitrogen). The ST8SIA mRNA was quantified using a SYBR Green Quantitative Real-time PCR mix (TaKaRa, Otsu, and Shiga, Japan) and normalized to GAPDH (glyceraldehyde-3-phosphate dehydrogenase). The sequences of the upstream and downstream primers were as follows: 5′-TAC TCT CTC TTC CCA CAG G-3′; 5′-GAC AAA GGA GGG AGA TTG C-3′ for ST8SIA1; 5′-GTG GTC TTC CTC ATC TTC G-3′; 5′-GAG GAG CCG TTT ATT ACA AC-3′ for ST8SIA2; 5′-ATT CTC TCA CCC AGG AAC TC-3′ and 5′-CAA TCC GAA CAC TAT TCT TG-3′ for ST8SIA3; 5′-CAA GAA CTG AGG AGC ACC-3′ and 5′-TTT CCA ACC TTC TAC ATT GTG-3′ for ST8SIA4; 5′-CCT TTG CCT TGG TGA CCT-3′ and 5′-CAT GGA CAG CAC CTT CAC T-3′ for ST8SIA5; 5′-CGG CAA GCA GAA GAA TAT G-3′ and 5′-GCT TTC CAC CTC GTA ACT C-3′ for ST8SIA6; 5′-CTC CTC CAC CTT TGA CGC TG-3′; 5′-TCC TCT TGT GCT CTT GCT GG-3′ for GAPDH. The expression level of the target genes was determined relative to GAPDH expression and calculated using the 2^−ΔΔCT^ method.

The expression of miR-146a/b was determined using a mirVana Quantitative Reverse Transcription Polymerase Chain Reaction (qRT-PCR) microRNA Detection Kit according to the manufacturer′s protocol (Ambion Inc., Austin, TX, USA) and normalized relative to U6-small nuclear RNA using the 2^−ΔΔCT^ method. For reverse transcription, 5 μg of total RNA was converted to cDNA using a TaqMan MicroRNA Reverse Transcription Kit (Fermentas) according to the manufacturer's protocol. The resulting cDNA was diluted 1:10 and used for PCR with 4 μL of miR-146a/b or U6 TaqMan primers and SYBR Green/Fluorescein qPCR Master Mix (Fermentas) with the ABI PRISM 7900HT Sequence Detection System (Applied Biosystems, Foster City, CA, USA). Next, 10 ng of total RNA was reverse transcribed using a High-Capacity cDNA Archive Kit (Applied Biosystems Inc., Foster City, CA) and then amplified on a ABI 7500 Real-Time PCR System (Applied Biosystems Inc., Foster City, CA). Real-time PCR was performed for 2 min at 95°C and then for 40 cycles of amplification for 20 s at 95°C and 1 min at 60°C.

### Western blot analysis

Whole cell proteins were electrophoresed under reducing conditions on a 10% polyacrylamide gel. The separated proteins were transferred to a polyvinylidene difluoride membrane. After the membranes were blocked with 5% skim milk in PBS containing 0.1% Tween 20 (PBST), they were incubated with the appropriate antibody (1:1000; Abcam, Cambridge, UK) overnight at 4°C and then incubated with peroxidase-conjugated anti-rabbit IgG (1:10000; GE Healthcare UK Ltd., Little Chalfont, U.K.). GAPDH was used as a control. Band intensity was evaluated using an ECL Western Blotting Substrate Kit (Amersham Biosciences, Buckinghamshire, UK) according to the manufacturer's protocol. The bands were analysed using LabWorks (ver4.6, UVP, BioImaging Systems).

### Transfection and luciferase activity assay

Mimics, antagomirs and negative control oligonucleotides for hsa-miR-146a/b were obtained from RiboBio Co., Ltd. (Shanghai, China). Plasmids containing WT Luc-ST8SIA4, mutant Luc-ST8SIA4, and lenti-miR-146a/b were synthesized. FTC cells were transfected using Lipofectamine 2000 reagent (Invitrogen, Carlsbad, CA) according to the manufacturer's instruction. Luciferase activity was measured 48 h after transfection using the Dual-Luciferase Reporter Assay System (Promega). Firefly luciferase activity was normalized to Renilla luciferase activity for each sample. The mean of the results from the cells transfected with the miR-control was set at 1.0. The data are presented as the mean value ± SD for three repeated experiments.

### Downregulation of ST8SIA4 by RNAi

FTC-133 cells were incubated in the appropriate antibiotic-free medium with 10% foetal bovine serum. The cells were then transferred to a 6-well plate prior to incubation in a CO_2_ incubator at 37°C to obtain 60–80% confluency. ST8SIA4 shRNA was mixed with Lipofectamine 2000. The cells were then harvested for further analysis. The transfected cells were cultured at 37°C for 6 h and then incubated with complete medium for an additional 24 h. Thereafter, the cells were harvested for further examination.

### Overexpression of ST8SIA4

The human ST8SIA4 coding sequences obtained from TaKaRa (Dalian, China) were inserted into the pEGFP-N2 vector (Invitrogen, Carlsbad, CA) at the EcoRI and XhoI sites. The cells were transfected with 5 μg of the target gene expression vector or empty vector (EV) in 100 mm dishes using PolyFect Transfection Reagent (Qiagen, Valencia, CA) according to the manufacturer's instructions. After 4 weeks of screening, the cell lines stably expressing ST8SIA4 (overexpression of ST8SIA4 in FTC-238 cells) and the corresponding empty vector (FTC-238/mock) were established and verified using western blotting.

### Cell proliferation assay (CCK8 assay)

For the proliferation assay, the transfected cells and negative control (NC) cells were seeded at 5 × 10^3^ cells/well in 96-well plates and incubated at 37°C for 4 days. A 10 μl aliquot of Cell Counting Kit-8 reagent (Dojindo, Japan) was added to the cells. Following incubation for 3 h, absorbance was measured at 450 nm using a spectrophotometer (Bio-Rad, USA).

### *In vitro* invasion assay

Cell invasion *in vitro* was demonstrated using 24-well Transwell units (Corning, NY, USA) with a 8 μm pore size polycarbonate filter coated with extracellular matrix gel (EC Matrix gel) (Millipore Chemicon, MA, USA) to form a continuous thin layer. The cells (3 × 10^5^) were harvested in serum-free medium containing 0.1% BSA and then added to the upper chamber. The lower chamber contained 500 μl DMEM. The cells were incubated for 24 h at 37°C in an incubator containing 5% CO_2_. After incubation, the cells on the upper surface of the filter were completely removed by wiping with a cotton swab. The filters were then fixed in methanol and stained with Wright-Giemsa stain. Cells that had invaded the Matrigel and reached the lower surface of the filter were counted using a light microscope at a magnification of 40×.

### Wound-healing assay

For the wound-healing assays, confluent monolayers of FTC-133 and FTC-238 cells were treated with 2 μg/ml of mitomycin for 2 h (Sigma-Aldrich). The transfected cells were seeded onto 6-well plates at 1 × 10^5^ cells/l and then cultivated in DMEM to maintain adherent cell growth for 6 h. Scratching (wounding) was performed using a 10 μl Eppendorf tip. The cells were cultured in 5% CO_2_ at 37°C for 24 h after they were washed in serum-free medium 3 times. Images were taken to determine the number of migrating cells between the scratches using Image-Pro Plus 6.0 software. The mean values and standard deviations were calculated for the intra-group comparisons. The experiment was repeated 3 times.

### Colony formation assays

The transfected cells were seeded in 6-well (100 cells/well) culture plates (BD Biosciences). The cells were incubated at 37°C with 5% CO_2_ for 7–12 days until visible colony formation was observed in the dish. Subsequently, the culture medium was removed, and the wells were washed twice with phosphate-buffered saline (PBS). The colonies were fixed with 10% methanol for 20 min, dried and stained with 0.1% crystal violet solution for 15 min. Each plate was then washed three times with 9% sodium chloride solution. Cell colonies with > 50 cells were counted and imaged.

### *In vivo* tumourigenicity assay

Athymic nude mice (5-week-old) were obtained from the Animal Facility of Dalian Medical University and provided with sterilized food and water. Approximately 1 × 10^7^ cells were injected subcutaneously into the right flank of each nude mouse. After palpable tumours were identified (approximately 4 weeks after tumour cell inoculation), the mice were sacrificed, and their tumours were isolated and weighed.

### Statistical analysis

The data are expressed as the mean ± standard deviation (SD). SPSS 17.0 software was used for the statistical analysis, and Student's *t-test* was used to determine the significance of differences among the examined groups. Normally distributed data were compared using a one-way ANOVA followed by the Student-Newman-Keuls test. A value of *p* < 0.05 was considered statistically significant.

## SUPPLEMENTARY MATERIALS FIGURES AND TABLES


