# Supplementary material for: miR-146a and miR-146b promote proliferation, migration and invasion of follicular thyroid carcinoma via inhibition of ST8SIA4
Source: Oncotarget. 2017 Mar 3;8(17):28028–41. doi: 10.18632/oncotarget.15885 (PMC5438628; doi:10.18632/oncotarget.15885)
Supplement: Supplementary file 1 [file oncotarget-08-28028-s001.pdf]

# miR-146a and miR-146b promote proliferation, migration and invasion of follicular thyroid carcinoma via inhibition of ST8SIA4

## Supplementary Materials

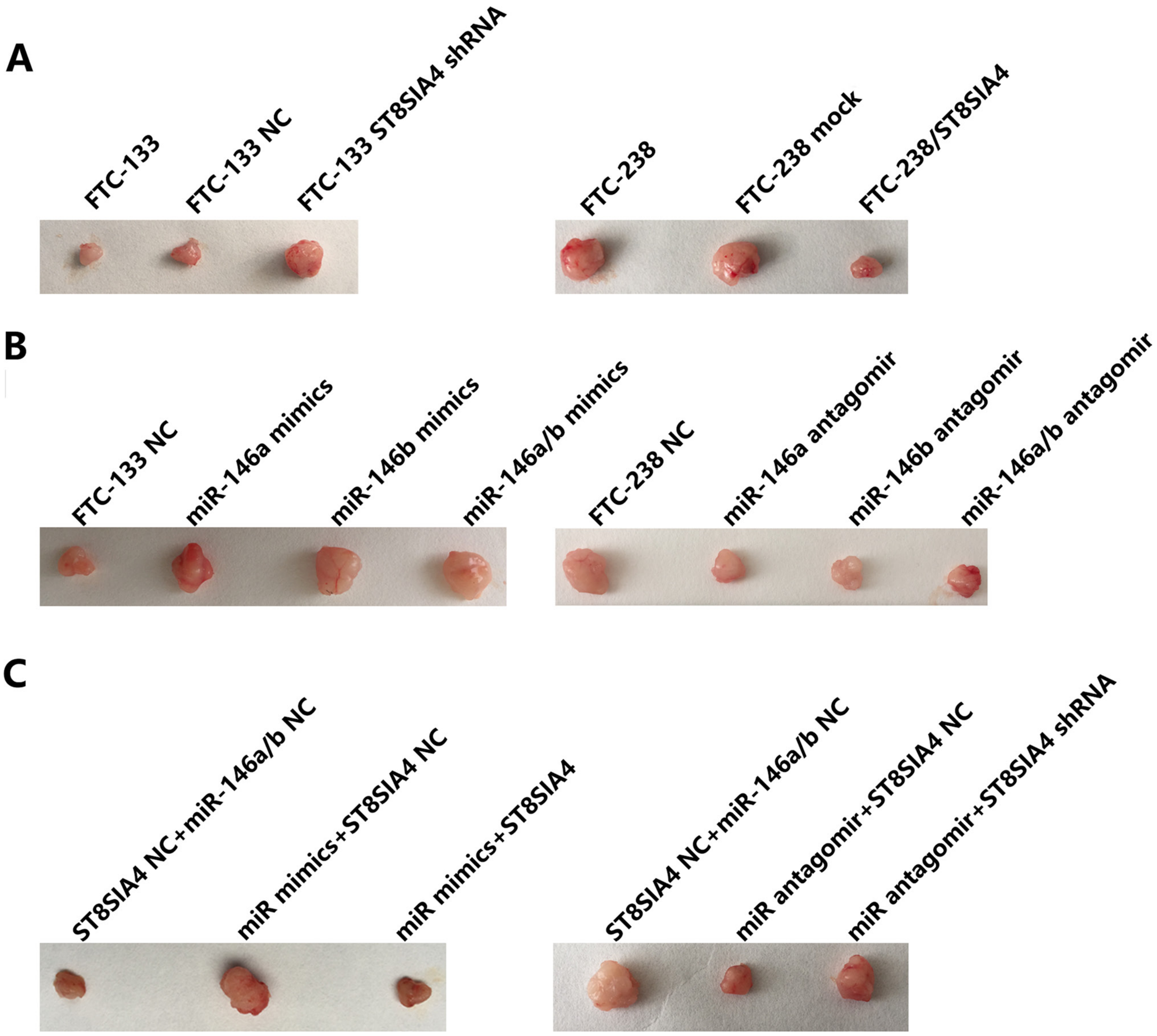


**Supplementary Figure 1: Tumours after transplantation, related to Figures 2, 4 and 5.** (**A**) (**B**) (**C**) Transfected cells were injected subcutaneously into nude mice; representative photograph of xenograft tumours (*$p < 0.05$)
